# RNA sequencing reveals lncRNA-mediated non-mendelian inheritance of feather growth change in chickens

**DOI:** 10.1007/s13258-022-01304-2

**Published:** 2022-09-10

**Authors:** Mohan Qiu, Chunlin Yu, Shiliang Zhu, Siyang Liu, Han Peng, Xia Xiong, Jialei Chen, Xiaosong Jiang, Huarui Du, Qingyun Li, Zengrong Zhang, Chaowu Yang

**Affiliations:** 1 Animal Breeding and Genetics Key Laboratory of Sichuan Province, Sichuan Animal Science Academy, 7 Niusha Road, 610066 Chengdu, China; 2 Farm Animal Genetic Resources Exploration and Innovation Key Laboratory of Sichuan Province, Sichuan Agricultural University, Xinkang Road, 610066 Chengdu, China

**Keywords:** Feather rate phenotype, Long non-coding RNAs, mRNA, Differentially expressed genes, cis-acting, Competing endogenous RNAs

## Abstract

**Background:**

Long non-coding RNAs (lncRNAs) play an essential role in biological processes. However, the expression patterns of lncRNAs that regulate the non-Mendelian inheritance feather phenotypes remain unknown.

**Objective:**

This study aimed to compare the expression profiles of lncRNAs in the follicles of the late-feathering cocks (LC) and late-feathering hens (LH) that followed genetic rules and the early-feathering hen (EH) and early-feathering cock (EC) that did not conform to the genetic laws.

**Methods:**

We performed RNA sequencing and investigated the differentially expressed lncRNAs (DElncRNAs) between the early- and late-feathering chickens, which function by cis-acting or participate in the competing endogenous RNA (ceRNA) network.

**Results:**

A total of 53 upregulated and 43 downregulated lncRNAs were identified in EC vs. LC, and 58 upregulated and 109 downregulated lncRNAs were identified in EH vs. LH. The target mRNAs regulated by lncRNAs in cis were enriched in the pentose phosphate pathway, TGF-β signaling pathway and Jak-STAT signaling pathway in EC vs. LC and were associated with the TGF-β signaling pathway, Wnt signaling pathway, p53 signaling pathway and Jak-STAT signaling pathway in EH vs. LH. In addition, the lncRNA-mediated ceRNA regulatory pathways of hair follicle formation were mainly enriched in the TGF-β signaling pathway, Wnt signaling pathway, melanogenesis, and calcium signaling pathways. The levels of ENSGALG00000047626 were significantly higher in the late-feathering chickens than in the early-feathering chickens, which regulated the expression of SSTR2 by gga-miR-1649-5p.

**Conclusion:**

This study provides a novel molecular mechanism of lncRNA’s response to the feather rate that does not conform to the genetic laws in chickens.

**Supplementary Information:**

The online version contains supplementary material available at 10.1007/s13258-022-01304-2.

## Introduction

The feather rate phenotype in modern chicken production is commonly used to identify cocks and hens (Fang et al. 2018). The early feathering (EF) and late feathering (LF) are sex-linked phenotypes. The sex chromosomes are homozygous (ZZ) in cock and heterozygous (ZW) in the hen. It was demonstrated that the K locus is located on the Z chromosome and is related to the LF phenotype; the k^+^ allele is recessive and contributes to the EF (Derks et al. 2018). Theoretically, the offspring of the late-feathering cocks (LC) and early-feathering hens (EH) should be LC and late-feathering hens (LH). However, in the actual production of commercial chickens, cocks and hens still show early and late-feather rate phenotypes. It was speculated that LH or LF might be related to RNA-mediated epigenetic and downstream gene regulation.

Long non-coding RNAs (lncRNAs) are a kind of non-coding RNAs with a length of over 200 nucleotides (Fatica and Bozzoni 2014; Guttman and Rinn 2012), which are involved in a series of biological processes by regulating gene expression and modifying chromatin. For instance, lnc1589, lnc3500, or lnc7831 regulate the Wnt signaling during feather regeneration in the chicken (Lin et al. 2018). In addition, lncFAM which is related to the growth in chickens regulated the expression of its target gene FAM48A via cis-expression (Li et al. 2020). The lncRNA can act as competing endogenous RNAs (ceRNAs), competitively binding microRNAs (miRNAs), and then affecting the regulation of miRNAs on downstream target genes (Pilyugin and Irminger-Finger 2014). Existing investigations revealed that LncIRS1 controls muscle atrophy via sponging miR-15 family to activate the IGF1-PI3K/AKT pathway (Li et al. 2019). Moreover, lncRNA Gm15290 sponges miR-27b to promote PPAR-induced fat deposition in mice. However, further analysis is required on whether lncRNAs competitively combine miRNAs and regulate the non-Mendelian inheritance of feather rate phenotype.

To identify the lncRNAs and lncRNA-associated ceRNA network associated with feather rate phenotype in chickens, we analyzed the differentially expressed lncRNAs (DElncRNAs) between the LC and LH that followed genetic rules (Mendelian inheritance) vs. the EC and EH from the same generation and should be also late-feathered but were actually early-feathered (non-Mendelian inheritance). Moreover, the target genes of DElncRNAs were predicted, and the function and pathways were analyzed. The DElncRNAs and target genes were verified by real-time quantitative PCR (qRT-PCR). This study aimed to provide the expression profiles and underlying mechanisms of these lncRNAs affecting early and late feathers of non-Mendelian inheritance in chickens.

## Materials and methods

### Hair follicle collection from experimental chickens

**Fig. 1:**
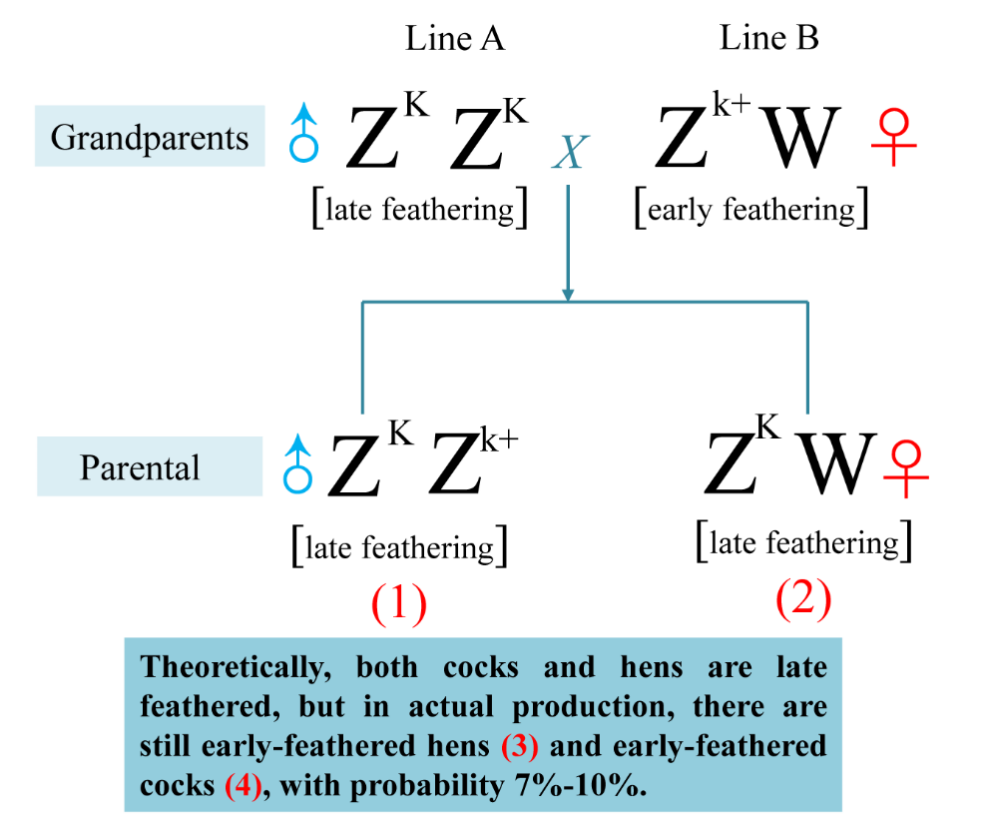
The genetic map of offspring from an early-feathering cock and a late-feathering hen. The samples were collected from the offspring, including four groups: late-feathering cock (LC) (1), and late-feathering hen (LH) (2) that follow genetic rules (Mendelian inheritance), and the early-feathering hen (EH) (3) and early-feathering cock (EC) (4) that should be also late-feathered but were actually early-feathered (non-Mendelian inheritance)

One-day-old chicks were procured from the Sichuan Dahen Animal breeding company. As shown in Fig. 1, the hair follicles of chickens were collected from four groups, including LC (1), LH (2), EH (3) and EC (4). The LC and LH were late-feathered chickens that followed genetic rules (Mendelian inheritance). The EC and EH were chickens from the same generation and should be also late-feathered but were actually early-feathered (non-Mendelian inheritance). Three chicks per group were used for the experiment. The animal care and ethical committee of the Sichuan Animal Science Academy granted the animal procedures in this study. All the experiments in this study were carried out based on the guidelines of the care and ethical use of laboratory animals.

### RNA extraction and transcriptome sequencing

The total RNA was isolated from the hair follicle samples using TRIzol reagent (Invitrogen) following the manufacturer’s instructions. The RNA integrity was detected by 1% agarose gel electrophoresis. NanoDrop 2000 spectrophotometer (Thermo Fisher Scientific) was used to check RNA purity and concentration. First, ribosomal RNA (rRNA) was removed using an EpicentreRibo-Zero™ rRNA Removal Kit (Epicentre, WI, USA). Second, RNA-sequencing libraries were generated under the instructions provided by the NEB Next Ultra™ Directional RNA Library Prep kit for Illumina (New England BioLabs, Inc.) Subsequently, the pooled libraries were sequenced on Hiseq X (Illumina, San Diego, CA, USA), using a chain-specific library construction strategy to count the number and types of lncRNAs.

### Data quality control and analysis

After discarding adapters and low-quality reads, the remaining high-quality clean reads were used for subsequent analyses. Thereafter, the clean read alignment was performed by tophat2 (Kim et al. 2013). Counting the number of gene reads and calculating FPKM were performed (Trapnell et al. 2010) by uniquely mapped reads.

### Identification of DElncRNAs from EC, LC, EH, and LH

DElncRNAs were identified using the DEG-seq software (Wang et al. 2010) for EC vs. LC and EH vs. LH. DElncRNAs of each comparison were identified with a threshold of log_2_|fold change| > 1 and p < 0.05.

### Target gene prediction and functional analysis

Prediction of DElncRNAs target genes was performed by cis-acting. For each lncRNA locus, the 100 kb neighboring protein-coding genes (without overlap) were identified as cis-acting target genes. The analysis of gene ontology (GO) terms and Kyoto Encyclopedia of Genes and Genomes (KEGG) pathways were performed by the KOBAS 2.0 server (Xie et al. 2011).

### Validation of DElncRNAs by qRT-PCR

To validate the expression of feather rate phenotype-related lncRNAs, three lncRNAs were selected for qRT-PCR. cDNA was then synthesized with the PrimeScript RT Reagent Kit (Takara, Japan). qRT-PCR was performed using the SYBR® Green PCR Master Mix with the SYBR Green PCR Reagent Kit (Yeasen, China). In the qRT-PCR assay, each sample set three repetitions. The expression of each sample was normalized against that of GAPDH via the 2 ^-ΔΔCT^ method (Livak and Schmittgen 2001). Supplementary Table 1 shows the sequences of primers.

### Dual-luciferase reporter assay

The potential targets were predicted by StarBase v3.0 and TargetScan and verified by a dual-luciferase reporter assay. The sequence of SSTR2 3′UTR, ENSGALG00000047626 3′UTR were obtained by complete gene synthesis (Shanghai Yingbio Technology, Co., Ltd, Shanghai, China). The specific restriction sites (XhoI and NotI) were added at both ends of the sequence, and the fragment was cloned into the pUC57 vector. The PCR products were then analyzed by 1% gel agarose electrophoresis and purified by the QIAquick PCR Purification Kit (Qiagen, Germany). The purified product and vector were digested with XhoI and NotI. In addition, the target gene fragments were inserted into a psiCHECK-2 vector (Promega, USA). Subsequently, the ligated PCR products were transformed into 100 µL of *Escherichia coli* DH5α, and the transformed cells were grown on an LB plate containing kanamycin and cultured at 37 °C overnight. Single colonies were picked for PCR confirmation and sequenced using universal primers to screen for positive clones. Following this, the plasmid was extracted, and positive clones were sequenced. The cell transfection was performed using Lipofectamine^™^ 2000 (Invitrogen) according to the manufacturer’s instructions. The grouping was as follows: gga-miR-1649-5p/miR-NC + psiCHECK-SSTR2/psiCHECK-SSTR2-mut (Group I) and gga-miR-1649-5p/miR-NC + psiCHECK-ENSGALG00000047626/psiCHECK-ENSGALG00000047626-mut (Group II). Three parallel holes were used for each group of cells. Promega Dual-Luciferase Reporter System (Promega, USA) was used to perform dual-luciferase assays.

### Statistical analyses

The SPSS 20.0 statistical software (SPSS, Inc., Chicago, IL, USA) was used for statistical analysis. The data were obtained from at least three independent samples and expressed as mean ± standard deviation (SD). Student’s t-test was used for statistical comparison. The p-value < 0.05 was considered statistically significant.

## Results

### Overview of the sequencing results

To study the molecular mechanisms of the non-Mendelian inheritance of feather rate phenotype, we analyzed DElncRNAs between the LC and LH that follow genetic rules vs. the EC and EH that were from the same generation and should be also late-feathered but were actually early-feathered (Fig. 1), by RNA sequencing. The three EC samples were marked as EC-1, EC-2, and EC-3. The LC, EH, and LH groups were labeled similarly. Twelve samples were used for subsequent sequencing. A total of 1,269,214,694 raw reads and 1,225,536,374 clean reads were generated from 12 samples. Subsequently, these data were mapped to the chicken genome; the mapping ratio for EC-1, EC-2, EC-3, EH-1, EH-2, EH-3, LC-1, LC-2, LC-3, LH-1, LH-2, and LH-3 were 91.16%, 91.33%, 91.10%, 91.30%, 90.50%, 91.16%, 91.08%, 90.92%, 91.48%, 91.52%, 90.57%, and 51.54%, respectively (Supplementary Table 2).

### Identification of DElncRNAs in EC vs. LC and EH vs. LH

**Fig. 2:**
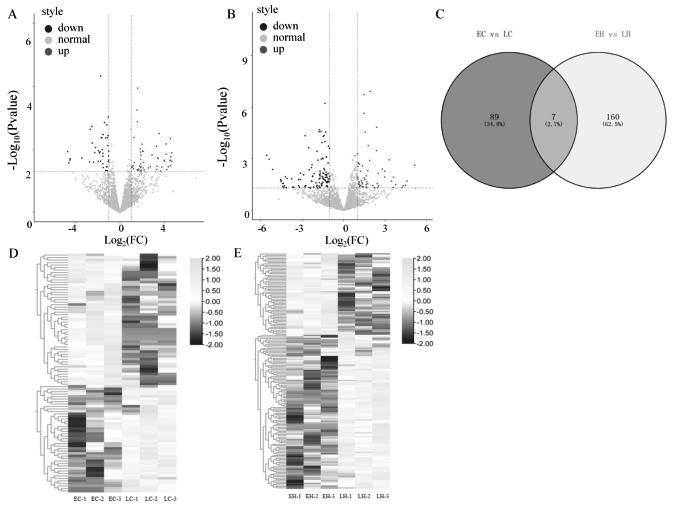
DElncRNAs analysis. (A) The expression pattern of DElncRNAs in EC vs. LC using DESeq2. The log2 Fold-Change ≥ 1 and p-value ≤ 0.05 were defined as upregulated genes, and log2 Fold-Change ≤-1 and p-value ≤ 0.05 were defined as downregulated genes. The upregulated lncRNAs were filled with red color, and the downregulated lncRNAs were filled with blue color. (B) The expression pattern of DElncRNAs in EH vs. LH. (C) The Venn diagram exhibits the intersection of DElncRNAs in EC vs. LC and EH vs. LH. (D) The hierarchical clustering heatmap of all the DElncRNAs in EC vs. LC. (E) The hierarchical clustering heatmap of all the DElncRNAs in EH vs. LH. LC (late-feathering cock) and LH (late-feathering hen) were chickens that followed genetic rules, and the EH (early-feathering hen) and EC (early-feathering cock) were chickens that should be also late-feathered but were actually early-feathered

To identify the potential DElncRNAs that were involved in early- and late-feathering chickens, a comparative analysis of the expression profiles of lncRNAs in chickens was performed. A total of 96 DElncRNAs were detected in EC vs. LC, and the up- and downregulated lncRNAs were 53 and 43, respectively (Fig. 2 A, Supplementary Table 3). Moreover, 167 DElncRNAs were identified in EH vs. LH, including 58 upregulated and 109 downregulated lncRNAs (Fig. 2B). There are seven common DElncRNAs between EH vs. LH and EC vs. LC, including ENSGALG00000048197, ENSGALG00000048627, ENSGALG00000054537, ENSGALG00000054359, ENSGALG00000053370, ENSGALG00000047626, and ENSGALG00000046870 (Fig. 2 C, Supplementary Table 4).

Moreover, we performed the hierarchical clustering of all the DElncRNAs in EC vs. LC and EH vs. LH. Heatmap for cocks and hens both show that the DElncRNAs could be significantly distinguished into early- and late-feather groups (Fig. 2D and E). The results suggested that compared with the same generation of late-feathering chickens that followed the genetic law, there were abnormal expression lncRNAs in early-feathering chickens that did not follow the genetic law. These lncRNAs might control the feather rate independently of the genetic law.

### Cis-acting of the feather rate phenotype-related lncRNAs

**Fig. 3:**
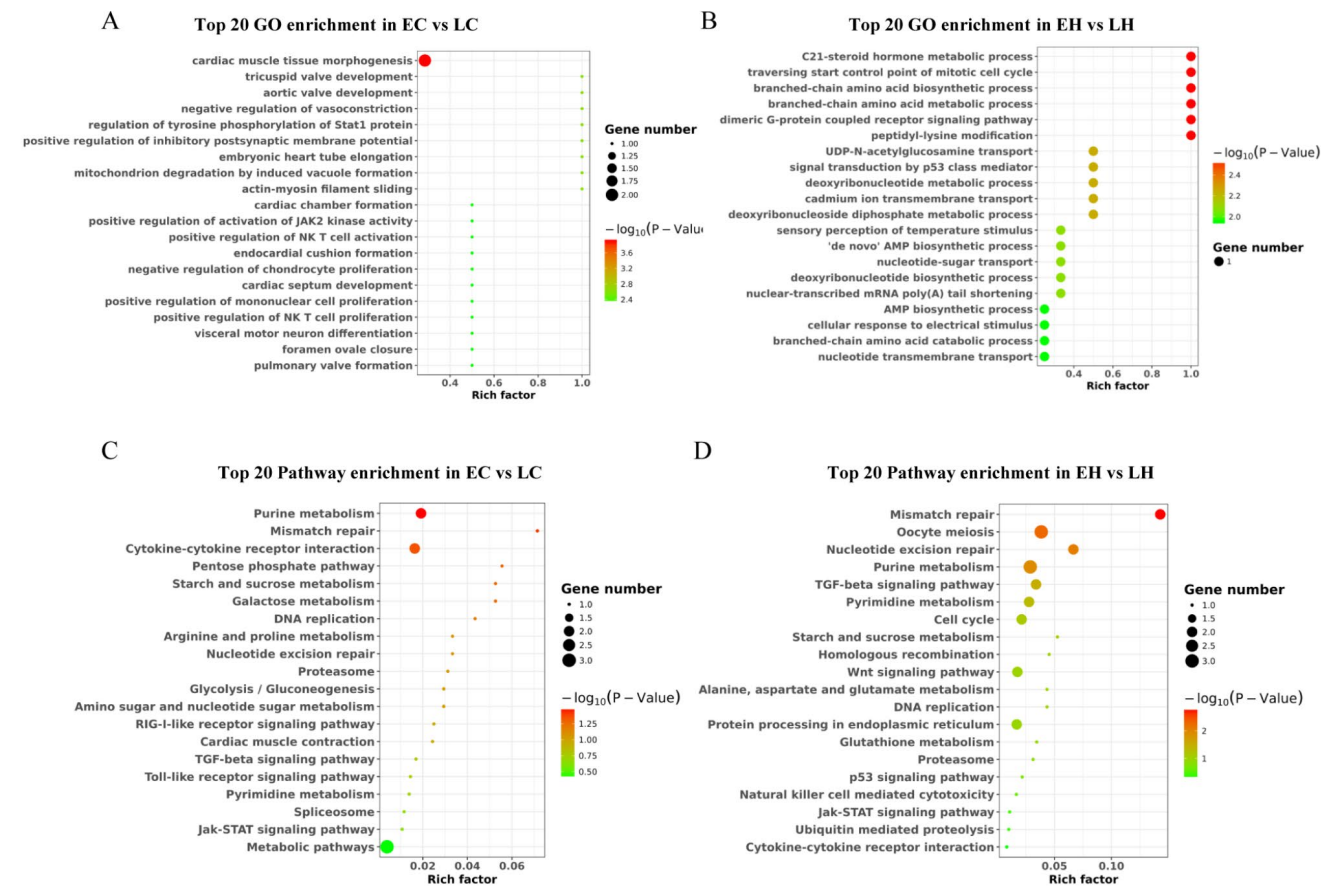
Search for the target mRNAs nearby the phenotype-related lncRNAs and functional analysis. (A) The top 20 enriched gene ontology (GO) terms for the target mRNAs nearby the phenotype-related lncRNAs in EC vs. LC. (B) The top 20 enriched GO terms for the target mRNAs nearby the phenotype-related lncRNAs in EH vs. LH. (C) The top 20 enriched Kyoto Encyclopedia of Genes and Genomes (KEGG) pathways for the target mRNAs nearby the phenotype-related lncRNAs in EC vs. LC. (D) The top 20 enriched KEGG pathways for the target mRNAs nearby the phenotype-related lncRNAs in EH vs. LH. LC (late-feathering cock) and LH (late-feathering hen) were chickens that followed genetic rules, and the EH (early-feathering hen) and EC (early-feathering cock) were chickens that should be also late-feathered but were actually early-feathered

To explore the function of these DElncRNAs, the cis-acting target mRNAs were analyzed with a threshold distance of 100 kb between mRNAs and lncRNAs, and 28 and 36 target mRNAs were screened in EH vs. LH and EC vs. LC, respectively. The GO term analysis revealed that these target mRNAs were implicated in the cardiac muscle tissue morphogenesis, and positive regulation of activation of JAK2 kinase activity in EC vs. LC (Fig. 3 A), and focused on C21 − steroid hormone metabolic process and traversing start control point of mitotic cell cycle in EH vs. LH (Fig. 3B). The KEGG pathway enrichment analysis showed that the pentose phosphate pathway, TGF-β signaling pathway and Jak-STAT signaling pathway were enriched significantly in EC vs. LC (Fig. 3 C). The TGF-β signaling pathway, Wnt signaling pathway, p53 signaling pathway and Jak-STAT signaling pathway were enriched significantly in EH vs. LH (Fig. 3D). Interestingly, Jak-STAT signaling pathway and TGF-β signaling pathway were the most prominent pathways in both EC vs. LC and EH vs. LH. These results demonstrated that the TGF-β and Jak-STAT signaling pathways possibly regulated feather rate phenotypes.

### Construction of lncRNA-miRNA-mRNA ceRNA network

**Fig. 4:**
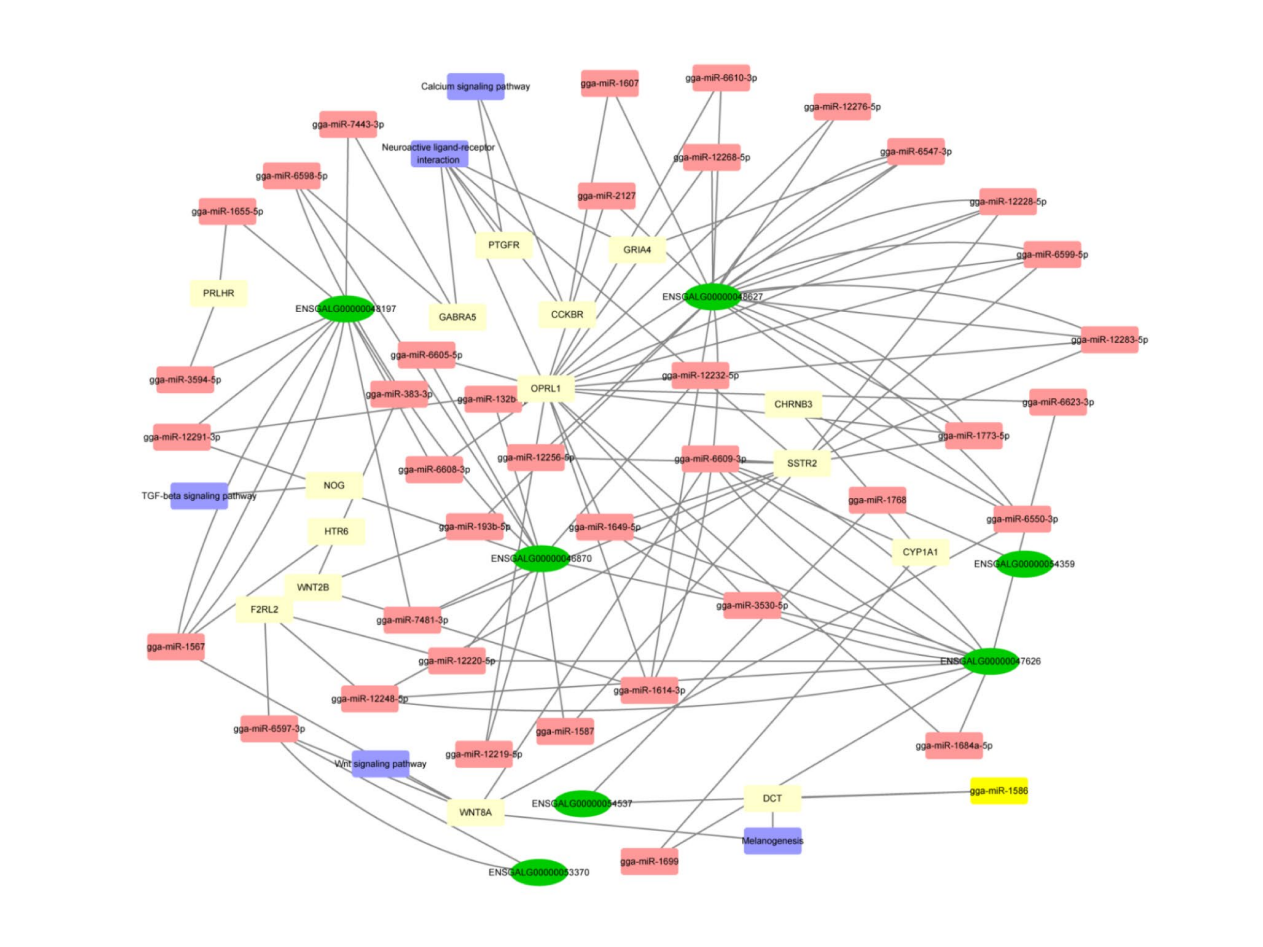
CeRNA network regulation model. Green indicates the lncRNAs, red indicates miRNAs, and yellow indicates mRNAs

To explore the lncRNA-mediated competing endogenous RNA (ceRNA) networks in the modulation of feather rate phenotype, we constructed the lncRNA-miRNA-mRNA ceRNA regulatory network (Fig. 4). In the network, WNT8A and F2RF2 might interact with lncRNA ENSGALG00000053370 mediated by miR-gga-miR-6597-3p. SSTR2 might interact with lncRNA ENSGALG00000047626 mediated by gga-miR-3530-5p. GABRA5 might interact with lncRNA ENSGALG00000046870 mediated by gga-miR-6598-5p.

**Fig. 5:**
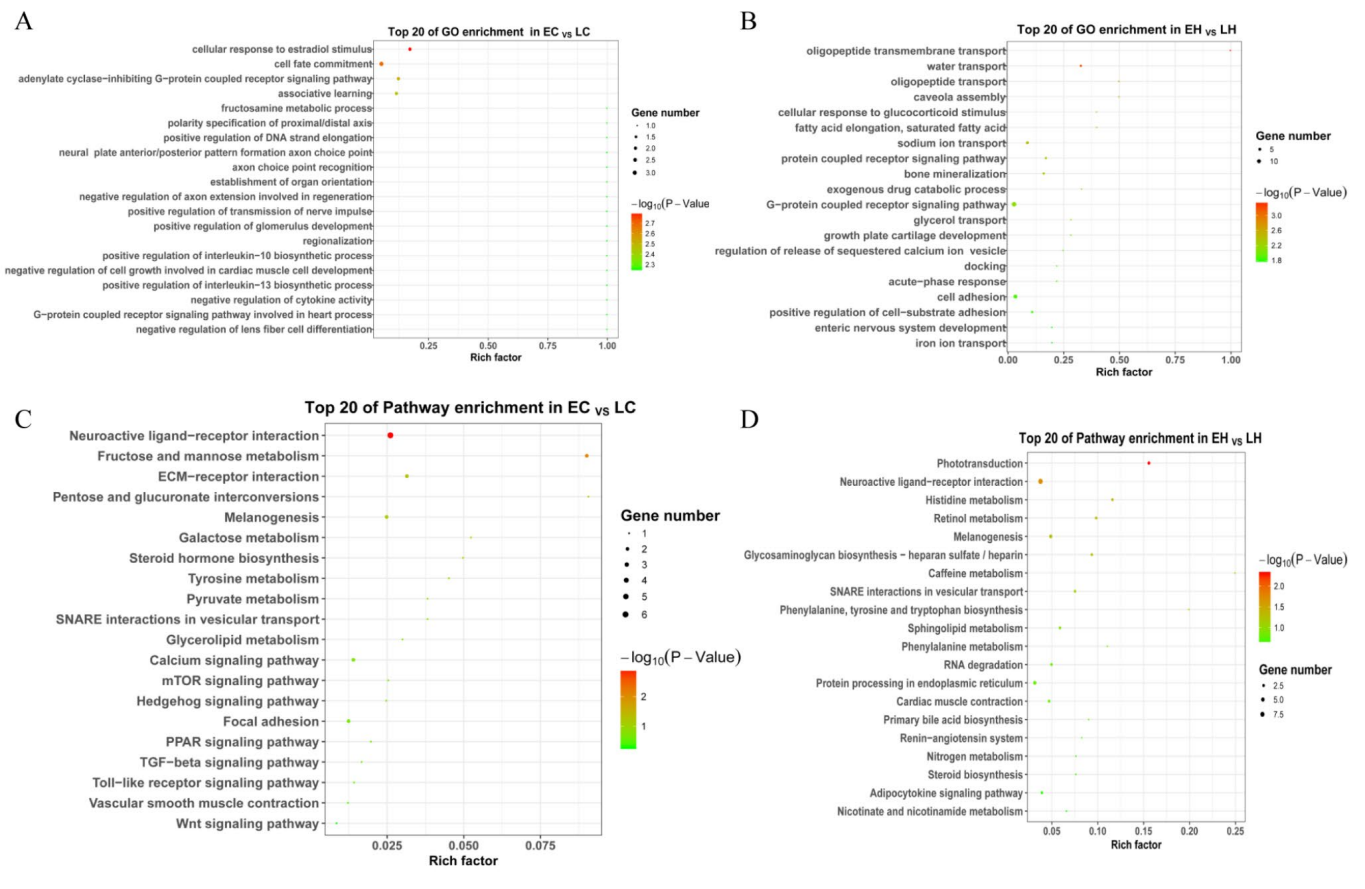
Functional enrichment analysis of the target mRNAs in ceRNA networks. (A) The top 20 enriched gene ontology (GO) terms for the target mRNAs in ceRNA networks in EC vs. LC. (B) The top 20 enriched GO terms for the target mRNAs in ceRNA networks in EH vs. LH. (C) The top 20 enriched Kyoto Encyclopedia of Genes and Genomes (KEGG) pathways for the target mRNAs in ceRNA networks in EC vs. LC. (D) The top 20 enriched KEGG pathways for the target mRNAs in ceRNA networks in EH vs. LH. LC (late-feathering cock) and LH (late-feathering hen) were chickens that followed genetic rules, and the EH (early-feathering hen) and EC (early-feathering cock) were chickens that should be also late-feathered but were actually early-feathered

### Analysis of functions and pathways of DElncRNAs via functioning as ceRNA

We performed GO and KEGG analyses to investigate the function and pathway of DElncRNAs. GO enrichment analysis showed target mRNAs focused on cell fate commitment and adenylate cyclase-inhibiting G-protein-coupled receptor signaling pathway in EC vs. LC (Fig. 5 A), and participated in G-protein-coupled receptor signaling pathway, oligopeptide transmembrane transport, and cell adhesion in EH vs. LH (Fig. 5B). KEGG analysis revealed that the mRNAs in the ceRNA networks were associated with the calcium signaling pathway, TGF-β signaling pathway, and Wnt signaling pathway in EC vs. LC (Fig. 5 C), and participated in steroid biosynthesis, histidine metabolism and adipocytokine signaling pathway in EH vs. LH (Fig. 5D). Interestingly, the KEGG pathway enrichment analysis showed that both groups were enriched in neuroactive ligand-receptor interaction, SNARE interactions in vesicular transport and melanogenesis pathway.

### Analysis of DElncRNAs in EC, LC, EH, and LH

**Fig. 6:**
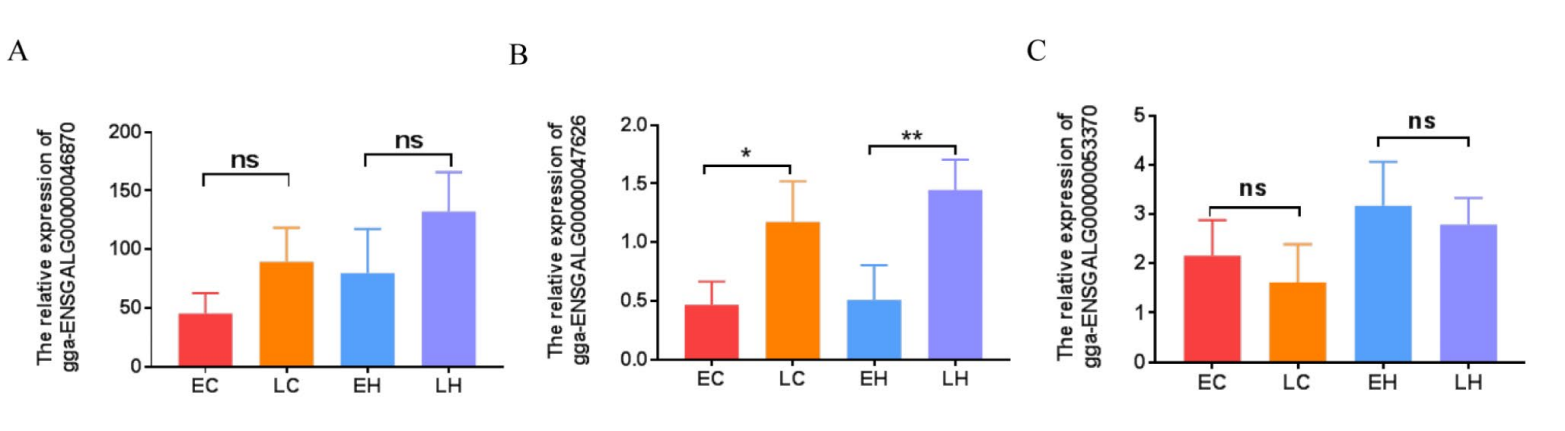
qRT-PCR validation of the three lncRNAs associated with the feather rate phenotype. (A) The relative expression of ENSGALG00000046870. (B) The relative expression of ENSGALG00000047626. (C) The relative expression of ENSGALG00000053370. LC (late-feathering cock) and LH (late-feathering hen) were chickens that followed genetic rules, and the EH (early-feathering hen) and EC (early-feathering cock) were chickens that should be also late-feathered but were actually early-feathered. *P < 0.05, **P < 0.01. Ns represents no significant difference

To screen for lncRNAs regulating feather rate phenotype, we performed qRT-PCR in potential DElncRNAs. We selected three DElncRNAs (ENSGALG00000053370, ENSGALG00000047626, and ENSGALG00000046870), which were differently expressed in EH vs. LH and EC vs. LC. Results showed that the level of ENSGALG00000047626 was significantly higher in LC than in EC, and was remarkably increased in LH relative to EH (Fig. 6B). However, ENSGALG00000053370 and ENSGALG00000046870 expression showed no significant difference in both EH vs. LH and EC vs. LC (Fig. 6 A, 6 C). Therefore, we hypothesized that ENSGALG00000047626 regulates feather rate phenotype via SSTR2.

### Validation of gga-mir-1649-5p targets

**Fig. 7:**
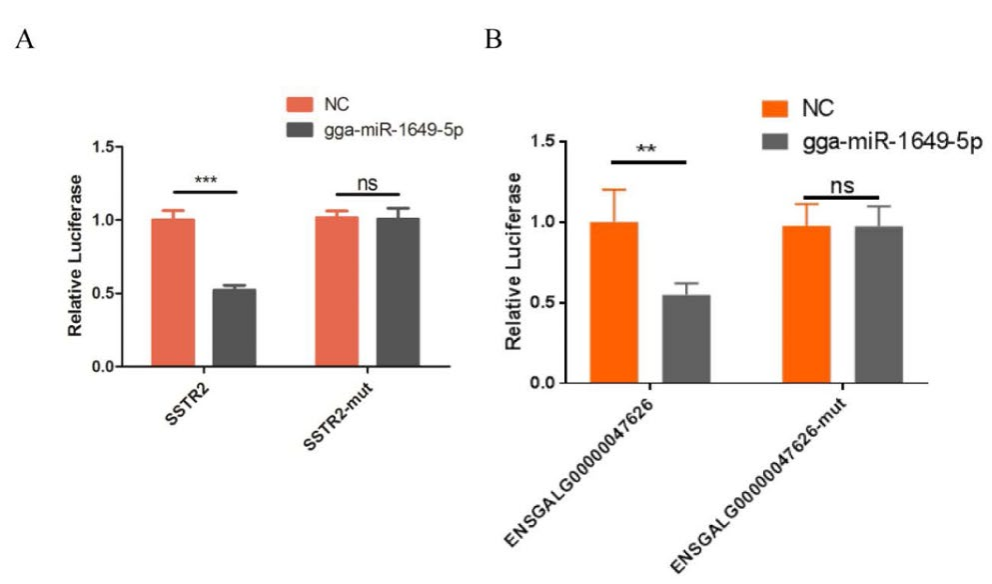
gga-miR-1649-5p targets ENSGALG00000047626 and SSTR2. Renilla luciferase activity was normalized to the firefly luciferase activity. (A) The gga-miR-1649-5p mimic was co-transfected with SSTR2 into the 293T cells. (B) The gga-miR-1649-5p mimic was co-transfected with ENSGALG00000047626 into the 293T cells. **P < 0.01, ***P < 0.001. Ns represents no significant difference

The prediction results of the TargetScan showed that gga-miR-1649-5p was likely to bind to ENSGALG00000047626 and SSTR2. To verify this, we carried out a dual-luciferase reporter assay. The results demonstrated that gga-miR-1649-5p significantly suppressed the luciferase activity of psiCHECK-SSTR2, whereas it did not affect the luciferase activity of psiCHECK-SSTR2-mut (Fig. 7 A). Moreover, gga-miR-1649-5p significantly inhibited luciferase activity of psiCHECK-ENSGALG00000047626 but not psiCHECK-ENSGALG00000047626-mut (Fig. 7B). It suggested that ENSGALG00000047626 might regulate the expression of SSTR2 by gga-miR-1649-5p to control the feather rate phenotype.

## Discussion

Sex identification is a feasible and vital strategy for breeding chickens commercially (Kreuzer et al. 2020). Feather rate phenotype is always used to identify the genders (Fang et al. 2018). However, the underlying molecular mechanism in the feather rate phenotype is unclear. In the present study, we performed RNA-sequencing analysis in EH vs. LH and EC vs. LC. A total of 96 and 167 DElncRNAs were detected in EC vs. LC and EH vs. LH, respectively, and 7 DElncRNAs were shared in EH vs. LH and EC vs. LC. KEGG enrichment analysis demonstrated that target mRNAs participating in the ceRNA network were mainly enriched in TGF-β signaling pathway, Wnt signaling pathway, and calcium signaling pathway. Moreover, ENSGALG00000047626 targeted SSTR2 and was significantly higher in late-feathering chickens than in early-feathering chickens. Interestingly, ENSGALG00000047626 could regulate the expression of SSTR2 by gga-miR-1649-5p.

Among the ceRNA regulatory pathways, we found a series of mRNAs that play crucial roles in the feather rate phenotype. KEGG enrichment analysis demonstrated that target mRNAs participating in the ceRNA network were mainly enriched in the Wnt signaling pathway, TGF-β signaling pathway and calcium signaling pathway. The Wnt/β-catenin pathway regulates pluripotent stem cell differentiation, organ development, and regeneration (Pang et al. 2021; Peng et al. 2021; Yin et al. 2022). Previous studies demonstrated that the Wnt pathway also plays a crucial role in hair follicles. For instance, the Wnt/β-catenin signaling pathway regulates feather follicle development and growth during the embryonic development of chicks (Xie et al. 2020). The expression of SNPs of the Wnt signaling pathway shows obvious differences in slow-feathering chickens and fast-feathering chickens (Qiu et al. 2020). WNT2, as a critical gene in the Wnt signaling pathway, could promote the hair follicle growth and development of skin and hair follicles in sheep (Tian et al. 2021). Moreover, the TGF-β signaling pathway is involved in feather formation. It was reported that all-trans-retinoic acid could suppress hair follicle growth via inhibiting proliferation and inducing apoptosis of dermal papilla cells partially via the TGF-β pathway (Nan et al. 2020). Ski, a repressor of TGF-β signaling, led to the inhibition of medulla formation (Tecalco-Cruz et al. 2018). In addition, keratin plays a crucial role in the hair growth cycle, and calcium ions significantly enhance keratin activity (Sharma and Kango 2021). The fast-feathering rate was primarily enriched in the calcium signaling pathway (Qiu et al. 2020). In this study, we found that the target mRNAs of DElncRNAs were significantly enriched in the TGF-β, Wnt, and calcium signaling pathways, suggesting that the DElncRNAs might mediate feather rate phenotype through these signaling pathways.

## Conclusion

This study revealed the differential expression pattern of lncRNAs between the early-feathering chickens that followed the genetic rules and the late-feathering chickens that did not follow the genetic rules. Multiple cis-target mRNAs of the DElncRNAs were enriched in the TGF-β signaling pathway and Jak-STAT signaling pathway. Moreover, we established the lncRNA-miRNA-mRNA ceRNA regulatory network. Our results provide novel insights into feather rate phenotype in chickens.

## Electronic supplementary material

Below is the link to the electronic supplementary material.

Supplementary Material 1

Supplementary Material 2

Supplementary Material 3

Supplementary Material 4

Supplementary Material 5

Supplementary Material 6
