# Supplementary material for: RNA sequencing reveals lncRNA-mediated non-mendelian inheritance of feather growth change in chickens
Source: Genes Genomics. 2022 Sep 10;44(11):1323–31. doi: 10.1007/s13258-022-01304-2 (PMC9569315; doi:10.1007/s13258-022-01304-2)
Supplement: Supplementary file 1 — Supplementary Material 1 [file 13258_2022_1304_MOESM1_ESM.docx]

**Supplementary Table 1.** Primer information.

| Gene | Sequence (5'-3') |
| --- | --- |
| gga-GAPDH-F | AGAAGGCTGGGGCTCATT |
| gga-GAPDH-R | TGCTAAGCAGTTGGTGGTG |
| gga-ENSGALG00000053370-F2 | CTGACCGCTCCCTACTACTG |
| gga-ENSGALG00000053370-R2 | TGCAAAGAAACAAGTGGCCA |
| gga-ENSGALG00000047626-F2 | CAGGGATCCTCTACCAGCAG |
| gga-ENSGALG00000047626-R2 | CGAACAACAGAAGAGCCGAG |
| gga-ENSGALG00000046870-F | TGACAAGCAACCAGGAACAA |
| gga-ENSGALG00000046870-R | ACCCAAAATTTGCAGTGCTGA |
| gga-ENSGALG00000053370-F2 | CTGACCGCTCCCTACTACTG |
