# Supplementary material for: RNA sequencing reveals lncRNA-mediated non-mendelian inheritance of feather growth change in chickens
Source: Genes Genomics. 2022 Sep 10;44(11):1323–31. doi: 10.1007/s13258-022-01304-2 (PMC9569315; doi:10.1007/s13258-022-01304-2)
Supplement: Supplementary file 2 — Supplementary Material 2 [file 13258_2022_1304_MOESM2_ESM.docx]

**Supplementary Table 2.** Sequencing statistics of the sequencing data.

| Sample | Raw reads | Raw base | Clean reads | Clean base | Reads Filter | Total mapped |
| --- | --- | --- | --- | --- | --- | --- |
| EC1 | 84977206 | 12746259632 | 82142254 | 12311260545 | 96.66% | 91.16% |
| EC2 | 96335978 | 14450029873 | 92945348 | 13929997321 | 96.48% | 91.33% |
| EC3 | 114982860 | 17246995915 | 111334938 | 16687202536 | 96.83% | 91.10% |
| EH1 | 103184602 | 15477304978 | 99298738 | 14881180317 | 96.23% | 91.30% |
| EH2 | 113366086 | 17004479513 | 109381872 | 16394310132 | 96.49% | 90.50% |
| EH3 | 119834564 | 17974736051 | 115499494 | 17311435074 | 96.38% | 91.16% |
| LC1 | 91455302 | 13717949350 | 88531544 | 13269171758 | 96.80% | 91.08% |
| LC2 | 109164960 | 16374327992 | 104494802 | 15660324810 | 95.72% | 90.92% |
| LC3 | 126802534 | 19019906418 | 122760902 | 18399047763 | 96.81% | 91.48% |
| LH1 | 91824178 | 13773280672 | 88844956 | 13315680125 | 96.76% | 90.52% |
| LH2 | 101964918 | 15294352648 | 98824042 | 14812901304 | 96.92% | 90.57% |
| LH3 | 115321506 | 17297793283 | 1.11E+08 | 16707854547 | 96.67% | 91.54% |
