# Supplementary material for: RNA sequencing reveals lncRNA-mediated non-mendelian inheritance of feather growth change in chickens
Source: Genes Genomics. 2022 Sep 10;44(11):1323–31. doi: 10.1007/s13258-022-01304-2 (PMC9569315; doi:10.1007/s13258-022-01304-2)
Supplement: Supplementary file 3 — Supplementary Material 3 [file 13258_2022_1304_MOESM3_ESM.docx]

**Supplementary Table 3.** Top ten most up- or downregulated differentially expressed lncRNAs.

| AccID | log_2_FC | Pvalue | FDR | Style |
| --- | --- | --- | --- | --- |
| ENSGALG00000047076 | 4.145 | 0.0067 | 0.7564 | up |
| ENSGALG00000049294 | 4.196 | 0.0440 | 0.9993 | up |
| ENSGALG00000052177 | 4.326 | 0.0362 | 0.9993 | up |
| ENSGALG00000051979 | 4.482 | 0.0231 | 0.9568 | up |
| ENSGALG00000048161 | 4.504 | 0.0333 | 0.9993 | up |
| ENSGALG00000054179 | 4.507 | 0.0222 | 0.9568 | up |
| ENSGALG00000053150 | 4.509 | 0.0046 | 0.5732 | up |
| ENSGALG00000046851 | 4.551 | 0.0173 | 0.9568 | up |
| ENSGALG00000047013 | 4.636 | 0.0240 | 0.9568 | up |
| ENSGALG00000050643 | 4.648 | 0.0129 | 0.8523 | up |
| ENSGALG00000050751 | -6.417 | 0.0000 | 0.0194 | down |
| ENSGALG00000049112 | -4.679 | 0.0117 | 0.8523 | down |
| ENSGALG00000049757 | -4.572 | 0.0271 | 0.9671 | down |
| ENSGALG00000048576 | -4.502 | 0.0220 | 0.9568 | down |
| ENSGALG00000054985 | -4.457 | 0.0200 | 0.9568 | down |
| ENSGALG00000047298 | -3.424 | 0.0188 | 0.9568 | down |
| ENSGALG00000048347 | -2.725 | 0.0250 | 0.9568 | down |
| ENSGALG00000053726 | -2.659 | 0.0023 | 0.5558 | down |
| ENSGALG00000054757 | -2.527 | 0.0019 | 0.5464 | down |
