# Supplementary material for: RNA sequencing reveals lncRNA-mediated non-mendelian inheritance of feather growth change in chickens
Source: Genes Genomics. 2022 Sep 10;44(11):1323–31. doi: 10.1007/s13258-022-01304-2 (PMC9569315; doi:10.1007/s13258-022-01304-2)
Supplement: Supplementary file 4 — Supplementary Material 4 [file 13258_2022_1304_MOESM4_ESM.docx]

**Supplementary Table 4.** The information of seven overlapping lncRNAs in EH vs. LH and EC vs. LC.

| AccID | log_2_FC | Pvalue | FDR | Style |
| --- | --- | --- | --- | --- |
| ENSGALG00000048197 (EC vs LC) | -1.53296 | 0.02854 | 0.992746 | down |
| ENSGALG00000048627 (EC vs LC) | -1.68074 | 0.004328 | 0.57317 | down |
| ENSGALG00000054537 (EC vs LC) | 1.034131 | 0.036059 | 0.999339 | up |
| ENSGALG00000054359 (EC VS LC) | 2.016372 | 0.011428 | 0.852327 | up |
| ENSGALG00000053370 (EC vs LC) | -1.15497 | 0.04718 | 0.999339 | down |
| ENSGALG00000047626 (EC vs LC) | -1.39117 | 0.001401 | 0.527929 | down |
| ENSGALG00000046870 (EC vs LC) | -1.50767 | 0.037876 | 0.999339 | down |
| ENSGALG00000048197 (EH vs LH) | 1.488359 | 0.012821 | 1 | up |
| ENSGALG00000048627 (EH vs LH) | 1.040448 | 0.01544 | 0.125851434 | up |
| ENSGALG00000054537 (EH vs LH) | -1.12402 | 0.001736 | 0.040138232 | down |
| ENSGALG00000054359 (EH vs LH) | 1.634913 | 0.043179 | 1 | up |
| ENSGALG00000053370 (EH vs LH) | -1.25757 | 0.022347 | 1 | down |
| ENSGALG00000047626 (EH vs LH) | -1.13064 | 0.019671 | 0.14315948 | up |
