# Supplementary material for: RNA sequencing reveals lncRNA-mediated non-mendelian inheritance of feather growth change in chickens
Source: Genes Genomics. 2022 Sep 10;44(11):1323–31. doi: 10.1007/s13258-022-01304-2 (PMC9569315; doi:10.1007/s13258-022-01304-2)
Supplement: Supplementary file 5 — Supplementary Material 5 [file 13258_2022_1304_MOESM5_ESM.docx]

Dear Editors:

I’m Chaowu Yang, and I come from the Sichuan Animal Science Academy of China. We would like to submit the enclosed manuscript entitled “**RNA sequencing reveals lncRNA-mediated non-Mendelian inheritance of feather growth change in chickens**”, which be wish to be considered for publication in **Genes & Genomics**. The manuscript is intended to be a full-length paper. We would like to declare that the work described was original research that has not been published previously, and not under consideration for publication elsewhere, in whole or in part. We declared no conflicts of interest in this study.

Long non-coding RNAs (lncRNAs) play an essential role in biological processes. However, the expression patterns of lncRNAs that regulate the non-Mendelian inheritance feather phenotypes remain unknown. This study aimed to compare the expression profiles of lncRNAs in the follicles of the late-feathering cocks (LC) and late-feathering hens (LH) that followed genetic rules and the early-feathering hen (EH) and early-feathering cock (EC) that did not conform to the genetic laws. We performed RNA sequencing and investigated the differentially expressed lncRNAs (DElncRNAs) between the early- and late-feathering chickens, which function by cis-acting or participate in the competing endogenous RNA (ceRNA) network. A total of 53 upregulated and 43 downregulated lncRNAs were identified in EC vs. LC, and 58 upregulated and 109 downregulated lncRNAs were identified in EH vs. LH. The target mRNAs regulated by lncRNAs in cis were enriched in the pentose phosphate pathway, TGF-β signaling pathway and Jak-STAT signaling pathway in EC vs. LC and were associated with the TGF-β signaling pathway, Wnt signaling pathway, p53 signaling pathway and Jak-STAT signaling pathway in EH vs. LH. In addition, the lncRNA-mediated ceRNA regulatory pathways of hair follicle formation were mainly enriched in the TGF-β signaling pathway, Wnt signaling pathway, melanogenesis, and calcium signaling pathways. The levels of ENSGALG00000047626 were significantly higher in the late-feathering chickens than in the early-feathering chickens, which regulated the expression of SSTR2 by gga-miR-1649-5p. This study provides a novel molecular mechanism of lncRNA’s response to the feather rate that does not conform to the genetic laws in chickens.

We deeply appreciate your consideration of this manuscript for publication in **Genes & Genomics**. If you have any questions, please do not hesitate to contact me at the address [**chaowuyang@163.com**](mailto:chaowuyang@163.com)**.**

Sincerely Yours,

Chaowu Yang
