# Supplementary material for: RNA sequencing reveals lncRNA-mediated non-mendelian inheritance of feather growth change in chickens
Source: Genes Genomics. 2022 Sep 10;44(11):1323–31. doi: 10.1007/s13258-022-01304-2 (PMC9569315; doi:10.1007/s13258-022-01304-2)
Supplement: Supplementary file 6 — Supplementary Material 6 [file 13258_2022_1304_MOESM6_ESM.pdf]

enago
www.enago.cn
voxtab
www.voxtab.com

CERTIFICATE OF EDITING

This is to certify that the paper titled RNA sequencing reveals lncRNA-mediated non-Mendelian inheritance of feather growth change in chickens commissioned to us has been edited for English language, grammar, punctuation, and spelling by Enago, an editing brand of Crimson Interactive Consulting Co. Ltd.

commisoned

bsi. ISO/IEC 27001 Information Security Management

ISO/IEC 27001:2013 Certified

bsi. ISO 9001 Quality Management

ISO 9001:2015 Certified

CRIMSON INTERACTIVE CONSULTING CO LTD 克里门森互动(北京)顾问有限公司 1101030093948

Issued by:

北京市海淀区中关村南大街甲6号铸诚大厦607室，邮编100086
Tel:+86-10-5158 1987

Disclaimer:The author is free to accept or reject our changes in the document after our editing. However, we do not bear responsibility for revisions made to the document after our edit on 22nd July, 2022.

English www.enago.com, www.voxtab.com, www.ulatus.com
Japan www.enago.jp, www.voxtab.jp, www.ulatus.jp
Brazil www.enago.com.br
Germany www.enago.de
Turkey www.enago.com.tr
China www.enago.cn
Korea www.enago.co.kr
Taiwan www.enago.tw

About Crimson:
Crimson Interactive Consulting Co. Ltd. provides English language editing, transcription, and translation services to individuals and corporate customers worldwide.
